# Some Reminders in Treatment and Diagnosis

**Published:** 1897-04

**Authors:** 


### Some Reminders in Treatment and Diagnosis.

A patient upon entering the office, the physician should notice carefully his gestures and movements. Notice his complexion, the condition of his skin, whether it be moist or dry, odor of perspiration, also the appearance of his eyes and teeth; a white, chalky color of either may indicate scrofula, syphilis, etc. Small, white teeth and wasted gums, phthisis; the characteristic appearance of the finger nails in phthisis is not to be overlooked. Puffs under the eyes may mean heart trouble or Bright's disease.

Notice how he protrudes his tongue, if he puts it out pointed, with sharp and quick jerks, it means nervousness. If, on the other hand, it is fat and flabby, passive constipation.

Inquire into his family history if you think his case in any way serious.

Never ask a patient "what's the matter?" ask him what he complains of?

Never suggest anything to your patient.

Examine his urine if the nature of the case leads you to suspect renal trouble. Note color, odor, etc. A light color indicates phosphates; if dark, it may indicate blood. A milky color will lead you to suspect pus or mucus.

Note its reaction and specific gravity. A high specific gravity indicates sugar, while a low specific gravity indicates albumen.

Temperament must be considered in the treatment of disease.

Whatever organ may be diseased endeavor to remove the cause and put the part to rest, and never prescribe a drug that masks the symptoms of a disease.

In the presence of numerous patients always look cheerful.

Never confirm your diagnosis on only one symptom.

Habit has a wonderful effect on the race. In treating old people respect habits and make haste slowly.

No effect is the result of a single cause.

Make elimination the key-note of your practice in the treatment of inflammatory diseases.

To sum up. The points to be carefully considered in the treatment and diagnosis of disease are: Race, hereditary tendencies, habits, age, education, superstition, religion, external surroundings, temperament, air, soil, water, humidity, etc., etc. —*Charlotte Med. Journal.*

